# Understanding Subjective Comparison of Memory to Peers: A Biopsychosocial Approach

**DOI:** 10.1093/geroni/igaa057.3299

**Published:** 2020-12-16

**Authors:** Allison Walden, Anna Robertson, Madeline Lag

**Affiliations:** University of Colorado Colorado Springs, Colorado Springs, Colorado, United States

## Abstract

Identifying factors that contribute to subjective reports of memory decline may have important implications for interpretation of objective memory performance. Research suggests that subjective memory decline is influenced by affective symptoms such as anxiety and stress that are subsequently associated with poorer performance on neurocognitive tests. Personality is an additional factor, as evidence demonstrates that neuroticism may predict subjective memory decline. More research is needed to understand how factors such as demographics, personality traits, and subjective health impact subjective memory. The present study applies a comprehensive, biopsychosocial approach to identify constructs that predict how individuals view subjective memory decline compared to others their age. Researchers used a national database, Midlife in the United States (MIDUS), due to its comprehensive and diverse sample (N = 3,291). Predictor variables pertained to demographics, personality traits, as well as physical and mental health self-reports, and the outcome variable captured subjective memory relative to one’s peers. A three-step hierarchical regression model indicated that demographic variables, personality traits, and self-evaluated health significantly contributed to 26.7% of the variance of self-evaluated memory compared to others their age, F(35, 2836) = 29.48, p < .001, with each block incrementally adding to the significance of the findings. Results suggest that subjective evaluation of physical health, personality traits, and demographic variables uniquely influence subjective memory decline relative to others of the same age. Awareness of how biopsychosocial factors may contribute to affective symptoms may have implications for the interpretation of neuropsychological testing results.

